# Human Hepatocytes in Experimental Steatosis: Influence of Donor Sex and Sex Hormones

**DOI:** 10.3390/biomedicines13112770

**Published:** 2025-11-12

**Authors:** Lena Seidemann, Carolin Marie Rohm, Anna Stilkerich, René Hänsel, Christina Götz, Daniel Seehofer, Georg Damm

**Affiliations:** 1 Department of Hepatobiliary Surgery and Visceral Transplantation, Clinic for Visceral, Transplant, Thoracic and Vascular Surgery, Leipzig University Medical Center, 04103 Leipzig, Germany; lena.seidemann@medizin.uni-leipzig.de (L.S.); anna.stilkerich@medizin.uni-leipzig.de (A.S.); daniel.seehofer@medizin.uni-leipzig.de (D.S.); 2 Saxonian Incubator for Clinical Translation (SIKT), Leipzig University, 04103 Leipzig, Germany; carolin.rohm@medizin.uni-leipzig.de (C.M.R.); christina.goetz@medizin.uni-leipzig.de (C.G.); 3 Institute for Medical Informatics, Statistics and Epidemiology (IMISE), Leipzig University, 04107 Leipzig, Germany; rene.haensel@sikt.uni-leipzig.de

**Keywords:** primary human hepatocytes, sex differences, hepatic steatosis, MASLD, sex hormones, 17b-estradiol, estrogen, testosterone, progesterone

## Abstract

**Background/Objectives**: Metabolic dysfunction-associated steatotic liver disease (MASLD) is a sexually dimorphic condition, with higher prevalence in men than in women. Sex differences in hepatic lipid metabolism and the modulatory role of sex hormones have been described but are still insufficiently understood. The aim of this study was to introduce the variables sex and sex hormones into a human in vitro model of hepatic steatosis. **Methods**: Primary human hepatocytes (PHHs) were isolated from male and female donors, treated with free fatty acids (FFA) to induce steatosis, and further exposed to physiological concentrations of estrogen, progesterone, or testosterone. Intracellular triacylglyceride (TAG) content, lipid droplet (LD) formation, FFA uptake, and very-low-density lipoprotein (VLDL) excretion were assessed. In parallel, the expression of lipid metabolism-related genes was quantified by qPCR. **Results**: FFA treatment induced comparable uptake and intracellular TAG storage in both sexes. However, female PHHs secreted approximately twice as many VLDL particles as male cells. Steatosis significantly increased expression of *LDLR*, *CPT2*, and *PLA1A* only in male PHHs. Sex hormones exerted distinct, sex-specific effects: estrogen reduced TAG accumulation in female PHHs; whereas testosterone reduced TAG in male but increased it in female PHHs after prolonged treatment. LD characterization confirmed sex- and hormone-dependent differences in lipid storage patterns. In male PHHs, progesterone promoted lipid storage and increased apoB-100 secretion, accompanied by reduced *LDLR* and *APOA5* expression, and testosterone increased the FFA-mediated *CPT2* even further. **Conclusions**: Sex and sex hormones distinctly shape hepatocellular lipid handling under steatotic conditions. While female PHHs demonstrated greater lipid excretion capacity, male PHHs exhibited stronger transcriptional responses. Sex-specific responses to estrogen and testosterone resembled clinical observations on sex hormone effects. These findings highlight the need to account for sex-specific differences in MASLD pathophysiology and therapeutic strategies.

## 1. Introduction

Metabolic dysfunction-associated steatotic liver disease (MASLD) is, at present, the most rapidly increasing cause of end-stage liver disease [1,2]. Because of its higher prevalence in men than in women, it has been coined a sexually dimorphic disease [3]. Indeed, the liver is a sexually dimorphic organ that shows sex-specific gene expression patterns [4]. A substantial proportion of the genes with a sex-biased expression is related to hepatic lipid metabolism [5,6]. Sex hormone signaling is presumed to be a driver of the sexually dimorphic hepatic gene expression [7]. Moreover, many clinical observations point to a regulatory role of sex hormones in MASLD pathophysiology [8]. Interestingly, they seem to exert differential effects on the two sexes in terms of hepatic steatosis. Protective effects of estrogen are assumed in women, since female MASLD prevalence increases after menopause, but can be mitigated by hormone replacement therapy [9]. Androgens show opposite effects in the two sexes, being protective for men but increasing the risk of MASLD in females [10,11].

Although sex and sex hormones are important variables in hepatic physiology and MASLD pathology, sex as a biological variable has not been given sufficient consideration in liver research in the past [9]. The inclusion of sex hormones in in vitro MASLD models was suggested as a “first step towards sex-specific [MASLD] models” [12]. Primary human hepatocytes (PHHs) derived from male and female human donors are deemed to represent the closest model to study sex dimorphism due to their high similarity with the in vivo setting [13]. In a previous study, our group has investigated sex-specific differences in PHHs in their expression levels of hepatic lipid metabolism genes and their responsiveness towards sex hormones. Our results underscored the importance of the consideration of cellular sex for investigations on the pathophysiological mechanisms of MASLD [14]. However, the applied model did not reflect cellular processes of hepatic steatosis.

In the current study, we incorporated the variables of sex and sex hormones into an in vitro model of hepatic steatosis by treating PHHs of different sex with free fatty acids (FFA) and physiological concentrations of estrogen, progesterone or testosterone. Lipid uptake, storage, and excretion, as well as the expression levels of genes involved in lipid metabolism, were analyzed with the emphasis on the influence of donor sex and sex hormone treatment.

## 2. Materials and Methods

### 2.1. Isolation of Primary Human Hepatocytes

Liver tissue specimens for PHH isolation were obtained from patients undergoing hepatic resection at Leipzig University Hospital. This study adhered to the principles of the Declaration of Helsinki and received approval from the Ethics Committee of the Medical Faculty of Leipzig University (protocol numbers: 422/21-ek, SMART-NAFLD study; 425/21-ek, MoLiSeTi project; and 322/17-ek, biobanking and use of primary human liver tissues). Written informed consent was obtained from all donors. Further details are provided in the Institutional Review Board Statement, and donor characteristics are listed in Table 1. PHHs were isolated from non-tumorous liver tissue using a two-step perfusion with EDTA and collagenase following established procedures [15]. Following isolation, the cell suspension was rinsed with phosphate-buffered saline (PBS; Gibco, Paisley, UK), and viable hepatocytes were quantified using the trypan blue exclusion method in a Neubauer chamber. Cells were subsequently resuspended in phenol red-free William’s Medium E (WME; Gibco, Paisley, UK), supplemented with 10% fetal bovine serum (FBS) superior (Sigma-Aldrich, St. Louis, MO, USA), 2mM L-glutamine, 15 mM HEPES, 1% nonessential amino acids (MEM NEAA 100X), 1 mM sodium pyruvate, 100 U/mL penicillin, and 100 µg/mL streptomycin (all Gibco, Paisley, UK), 40 U/mL insulin (Eli Lilly and Company, Indianapolis, IN, USA), and 1 µg/mL dexamethasone (JENAPHARM, Jena, Germany) and then transferred to culture.

### 2.2. Cell Culture, Sex Hormone Treatment and Steatosis Induction

Culture dishes were pre-coated with a collagen type I-based extracellular matrix supplemented with 5% phenol red-free Matrigel (Corning Inc., Corning, NY, USA) following established procedures [16]. PHHs were seeded at a density of 500,000 cells per well in 24-well plates using PHH culture medium. Cells were allowed to attach for 4 h at 37 °C and 5% CO_2_ in a humidified incubator, after which non-adherent cells and debris were removed by washing with PBS. Fresh PHH medium was then added, and cultures were equilibrated overnight. Hormone treatment was initiated the next day and conducted in a similar manner to that described in our recent work [14]. Briefly, cells were washed twice with PBS and the medium was replaced either with control medium (in the same composition as PHH culture medium, but with only 5% FBS, and without dexamethasone and insulin) or with control medium containing one of the following hormones: 10 nM 17β-estradiol (E2758), 70 nM progesterone (P8783), or 40 nM testosterone (T1500) (all from Sigma-Aldrich, St. Louis, MO, USA). In vitro induction of steatosis was achieved on the basis of the model of Gómez-Lechón et al. [17]. After 24 h of incubation with either hormone containing or control medium, the cell medium was replaced by a 0.6 mM FFA-containing medium (oleic and palmitic acid at a 2:1 ratio) with or without added hormones in the above indicated concentrations. The FFA medium was prepared from control medium containing 0.2 mM sodium palmitate, 0.4 mM oleic acid (both purchased from Sigma Aldrich, St. Louis, MO, USA), and 0.3% methanol (Th. Geyer GmbH & Co. KG, Renningen, Germany). The cell medium was renewed every 24 h, and steatotic treatment with and without hormones lasted for a further 48 h. For each condition, PHHs of each donor were cultured and measured in two independent technical replicates.

### 2.3. TAG Assay

For quantitative analysis of TAG content, 5% NP40 (tergitol NP 40 Cell Lysis Buffer, Sigma Aldrich (St-Louis, MO, USA)) was added to cells after two washes. Cells were scraped off and heated up to 100 °C two times for 2 min followed by centrifugation at 4 °C for 5 min at 10,000× *g*. For analysis, Triglyceride Quantification Assay Kit (abcam, Cambridge, UK) was used according to the manufacturer’s instructions. Fluorescence was measured at Ex/Em 535/587 nm in a microplate reader (Synergy H1, BioTek, Winooski, VT, USA).

### 2.4. FFA Assay

Supernatants from FFA-treated PHHs were collected, centrifuged at 200× *g*, 5 min, 4 °C, and the residual FFA content was quantified using the Free Fatty Acid Assay Kit (Sigma-Aldrich, St. Louis, MO, USA). Apart from preparing standards and samples in steatosis medium (without FFA and methanol) instead of the provided assay buffer, the protocol was carried out according to the manufacturer’s instructions. Absorbance was recorded at 570 nm using a microplate reader.

### 2.5. ApoB-100 Assay

ApoB-100 is an apolipoprotein that is part of the hydrophilic surface of very-low-density lipoproteins (VLDL). Each VLDL particle contains one apoB-100 protein. Thus, measurement of apoB-100 in the cell culture supernatant was used to quantify lipid excretion. The supernatants at respective time points were collected, centrifuged at 200× *g*, 5 min, 4 °C, and stored at −80 °C until further analysis. ApoB-100 analysis was performed with Human apoB-100 ELISA Kit (EH35RB) purchased from Thermo Fisher Scientific (Waltham, MA, USA). The assay was performed according to the manufacturer’s instructions. Due to technical difficulties, no measurements could be obtained for the 48 h values of PHHs treated with FFA, FFA + estrogen, and FFA + progesterone of one male donor.

### 2.6. BCA Assay

Total protein contents were measured as a surrogate parameter for cell quantity for normalization purposes. For sample preparation, cells were washed and detached with a cell scraper and lysis buffer (50 mM Trizma HCl, 0.5% Triton X-100, 0.1% sodium dodecyl sulfate (all purchased from Sigma Aldrich, St-Louis, MO, USA) in PBS) was added. Plates were frozen at −20 °C overnight. The next morning, cells were thawed, mixed by pipetting up and down and sonicated. After centrifugation at 10,000× *g* for 10 min, supernatants were retained for measuring. A standard dilution series with BSA (bovine serum albumin, Sigma Aldrich, St-Louis, MO, USA) was prepared. Standards and samples were measured in duplicates. A reaction mix of 4% CuSO_4_ in BCA reagent A (both purchased from Sigma Aldrich, St-Louis, MO, USA) in a 1:50 ratio was added and plates were incubated at 37 °C for 30 min in the dark. Absorption was measured at 550 nm in a plate reader. Furthermore, cell viability was monitored by XTT assay according to the manufacturer’s instructions (Cell Proliferation Kit II by Sigma Aldrich, Merck KgaA, Darmstadt, Germany; for further information and results, see Figure S1 in Supplementary Materials).

### 2.7. RNA Isolation, Reverse Transcription, and qPCR Analyses

For RNA isolation, PHHs were detached after at least two washing steps and lysed in 200 µL RNA-Solv reagent per well using a cell scraper. The lysates were transferred into sterile RNase-free tubes and stored at −80 °C until use. RNA was extracted from thawed samples following the single-step procedure of Chomczynski and Sacchi [18]. The RNA pellet was dissolved in RNase-free water (Qiagen, Venlo, The Netherlands), and concentration, quality, and purity were assessed with a NanoDrop 2000 spectrophotometer (Thermo Fisher Scientific, Waltham, MA, USA). Reverse transcription was carried out using QuantiTect Reverse Transcription Kit (Qiagen, Venlo, The Netherlands) in accordance with the manufacturer’s protocol. Primers for qPCR were obtained from Qiagen or designed as intron-spanning, gene-specific primers using Primer3 software; details are listed in the Supplementary Materials (Table S1). Primer efficiency was determined from cDNA serial dilution curves, and specificity was confirmed by melt curve analysis and gel electrophoresis. qPCR reactions were run in duplicate with 20 ng cDNA per well using the QuantiNova^®^ SYBR^®^ Green PCR Kit (Qiagen, Venlo, The Netherlands) on a 7500 Real-Time PCR System with v2.0.6 software (Applied Biosystems, Foster City, CA, USA). The thermal profile consisted of an initial activation step at 95 °C for 5 min, followed by 40 cycles of 10 s denaturation at 95 °C and 30 s annealing/extension at 60 °C or 62 °C. Relative gene expression was determined according to the method of Taylor et al., with normalization to the average Cq values of the untreated controls of all donors (0 h, prior hormone treatment) and the geometric mean expressions of the three reference genes (RPL13A, EEF2, and RPS18). Statistical analysis was performed on log_2_-transformed expression values [19].

### 2.8. Fluorescence Staining, Microscopy and Imaging Analysis

For later microscopy, PHHs were cultured on coated glass coverslips (12 mm Ø, thickness no. 1; Th. Geyer GmbH & Co. KG, Renningen, Germany). Then, PHHs were washed twice with PBS, fixed with 4% paraformaldehyde solution for 10 min after the respective incubation times, and washed three times with PBS again. Cell nuclei were visualized with DAPI (Sigma-Aldrich, St. Louis, MO, USA) and cell membranes with Phalloidin iFluor 555 (Abcam, Cambridge, UK), both diluted in 0.2% BSA/PBS to a concentration of 1 μg/mL. Lipid droplets (LD) were stained with BODIPY™ 493/503 (Thermo Fisher Scientific, Waltham, MA, USA), which was used in a concentration of 25 μg/mL, diluted in 0.2% BSA/PBS. All stainings were performed for one hour at room temperature in the dark. After staining, coverslips were mounted with 10 µL of Mowiol 4-88 (Carl Roth, Karlsruhe, Germany) and stored at 4 °C in the dark. Images were recorded with a Keyence BZ-X800 fluorescence microscope (Keyence, Osaka, Japan) using a 60× objective. Z-stack recordings, with a thickness of 0.1 μm, were detected at ex/em 555/585 (Phalloidin), 405/435 (DAPI) 488/518 (Bodipy). A total of three images per condition were recorded from different areas of the slides, and middle layers were selected manually for imaging analysis. LD areas were segmented using Fiji v2.16.0 [20] with a StarDist v0.9.1 plugin [21]. LD counts and areas for each condition were analyzed with Python v3.12.10 [22] and visualized as particle size distribution plots using Seaborn module v0.13.2 [23].

### 2.9. Statistical Analysis

Statistical analyses were performed using GraphPad Prism 8 (San Diego, CA, USA). Differences between independent groups were analyzed with an unpaired, two-tailed *t* test. Differences between paired samples were analyzed with a paired *t* test (two-tailed). Statistical significance was assumed at *p* < 0.05.

## 3. Results

### 3.1. Male and Female PHHs Do Not Show Differences in Lipid Uptake or Storage, but in VLDL Excretion

**Figure 1:**
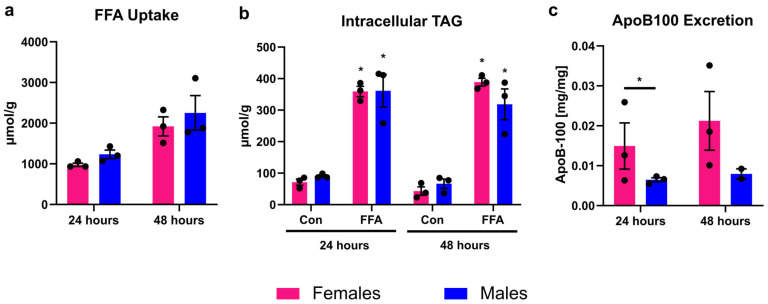
Sex-dependent analysis of lipid uptake, storage, and excretion. Primary human hepatocytes (PHHs) from female and male donors were cultured with 0.6 mM free fatty acids (FFA) and without (Con). (**a**) FFA uptake was determined by subtracting the FFA content measured in the cell culture supernatants after 24 and 48 h from the applied FFA concentrations. (**b**) Intracellular TAG content was analyzed. * Indicates statistically significant differences between FFA-treated and control PHHs of the same sex. (**c**) Very-low-density lipoprotein (VLDL) excretion from FFA-treated PHHs was determined by apoB-100 assay. * Indicates statistically significant differences between PHH of different sex. Measurements were normalized to protein content. Individual donor values are displayed as dots; bar graphs represent means ± SEM.

PHHs treated with FFA took up about 1000 µmol FFA per 24 h and g protein, which led to a significant increase in intracellular TAG content compared to control (Figure 1). FFA uptake and intracellular TAG storage did not differ between PHHs of different sex. Lipid excretion in the form of VLDL was measured by apoB-100 assay, since each VLDL particle carries one apoB-100 protein. The amount of excreted VLDL particles was twice as high in female than in male PHHs. This difference was significant at 24 h but did not reach statistical significance at 48 h (*p* = 0.0524, unpaired two-tailed *t* test). The 48 h values might indicate a trend in the same direction as the 24 h measurements. But it is important to note that apoB-100 values after 48 h were only available from two male donors due to technical difficulties.

### 3.2. Only Male PHHs Show Increased Lipid Metabolism Gene Expression After Induction of Steatosis

**Figure 2:**
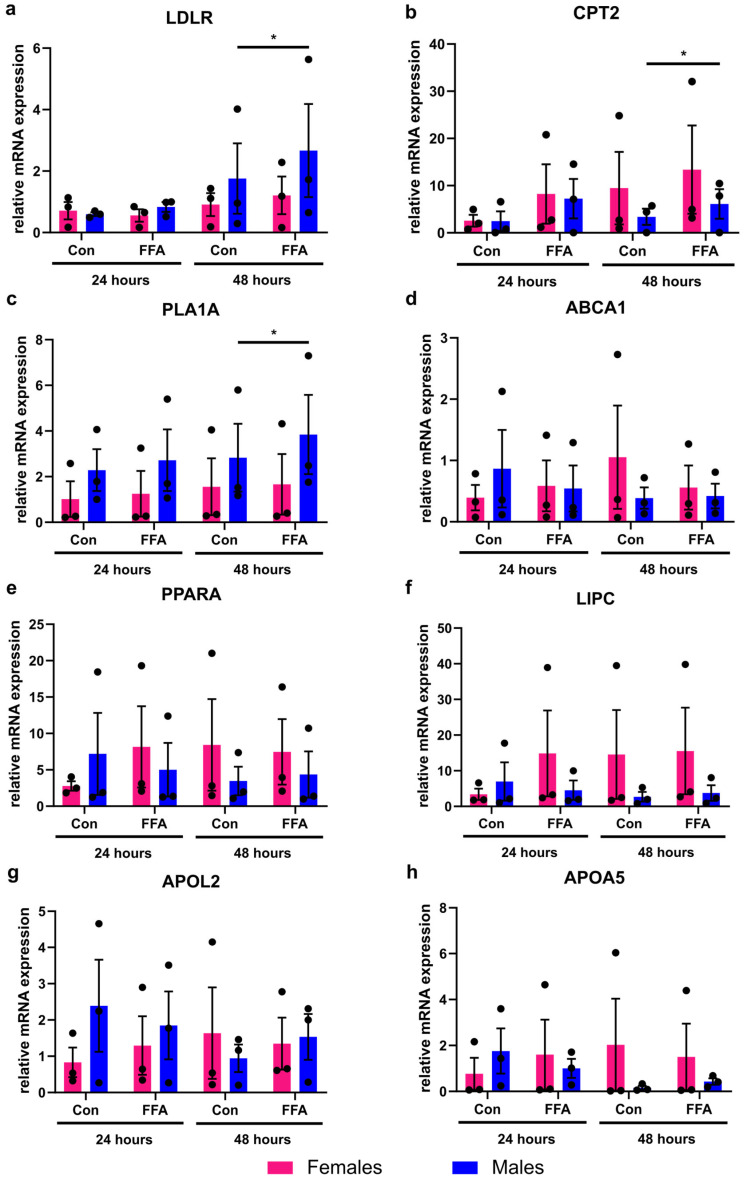
Influence of steatosis induction on lipid metabolism gene expression in PHHs of different sex. Primary human hepatocytes (PHHs) from female and male donors were cultured with 0.6 mM free fatty acids (FFA), and mRNA expression levels of (**a**) low-density lipoprotein receptor (*LDLR*), (**b**) carnitine palmitoyltransferase 2 (*CPT2*), (**c**) phospholipase A1 member A (*PLA1A*), (**d**) ATP-binding cassette, sub-family A, member 1 (*ABCA1*), (**e**) peroxisome proliferator-activated receptor alpha (*PPARA*), (**f**) hepatic lipase (*LIPC*), (**g**) apolipoprotein L2 (*APOL2*), and (**h**) apolipoprotein A-V (*APOA5*) were analyzed by RT-qPCR. Individual relative gene expression values per donor are displayed as dots; bar graphs represent arithmetic means ± SEM. Statistical analyses were performed on log_2_-transformed expression values, *n* = 3 per sex, *p* < 0.05 (*).

The mRNA expression analysis of eight different genes, associated with lipid metabolism, showed that significant changes induced by FFA treatment could be observed in male PHHs only (Figure 2). In female PHHs, steatosis induction did not lead to any significant increase in the measured lipid metabolism genes. Although individual female donors also showed increased CPT2 expression, variability between donors prevented statistical significance. In male PHHs, the mRNA expression levels of low-density lipoprotein receptor (*LDLR*), carnitine palmitoyltransferase 2 (*CPT2*), and phospholipase A1 member A (*PLA1A*) increased significantly after 48 h of FFA treatment.

### 3.3. Sex Hormones Have Sex-Specific Influence on Lipid Storage and Excretion

**Figure 3:**
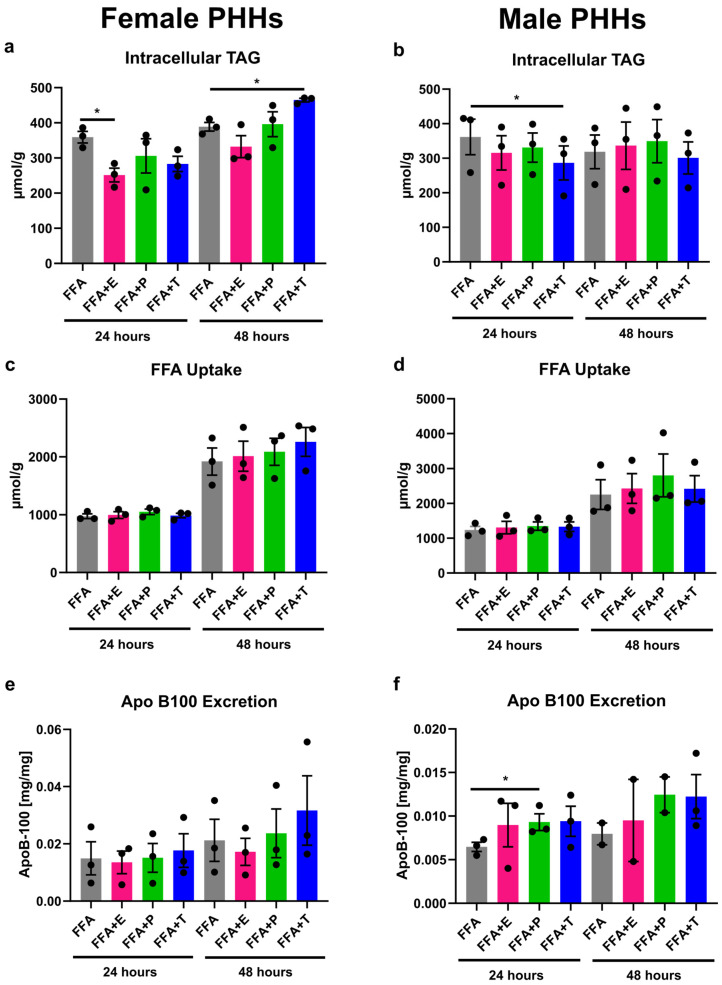
Influence of sex hormones on lipid uptake, storage, and excretion in PHHs of different sex. Primary human hepatocytes (PHHs) from female and male donors were cultured with 0.6 mM free fatty acids (FFA) with or without addition of 10 nM 17β-estradiol (E), 70 nM progesterone (P), or 40 nM testosterone (T). Intracellular TAG content was analyzed by TAG-assay in female (**a**) and male (**b**) PHHs. FFA uptake was determined by subtracting the FFA content measured in the cell culture supernatants after 24 and 48 h from the applied FFA concentrations to female (**c**) and male (**d**) PHHs. Very-low-density lipoprotein (VLDL) excretion from FFA-treated PHHs was determined by apoB-100 assay from female (**e**) and male (**f**) PHHs. Individual donor values are displayed as dots; bar graphs represent means ± SEM. * Indicates statistically significant differences compared to FFA alone.

When steatosis induction in PHHs was combined with sex hormone treatment, differential influences of hormones on PHHs from different sexes could be observed (Figure 3). Estrogen addition led to a significant decrease in intracellular TAG storage in female PHHs after 24 h. In male PHHs, testosterone significantly reduced TAG content after 24 h, whereas reductions after estrogen and progesterone treatment did not reach significance. A longer treatment of testosterone in female PHHs for 48 h, on the other hand, resulted in an increased intracellular TAG content. While FFA uptake was not influenced by sex hormones, progesterone increased the VLDL excretion in male PHHs after 24 h. Under all conditions, XTT assay measurements indicated stable cell activity (Figure S1 in Supplementary Materials).

### 3.4. Sex Hormones Influence Lipid Metabolism Gene Expression Sex-Specifically Under Steatotic Conditions

**Figure 4:**
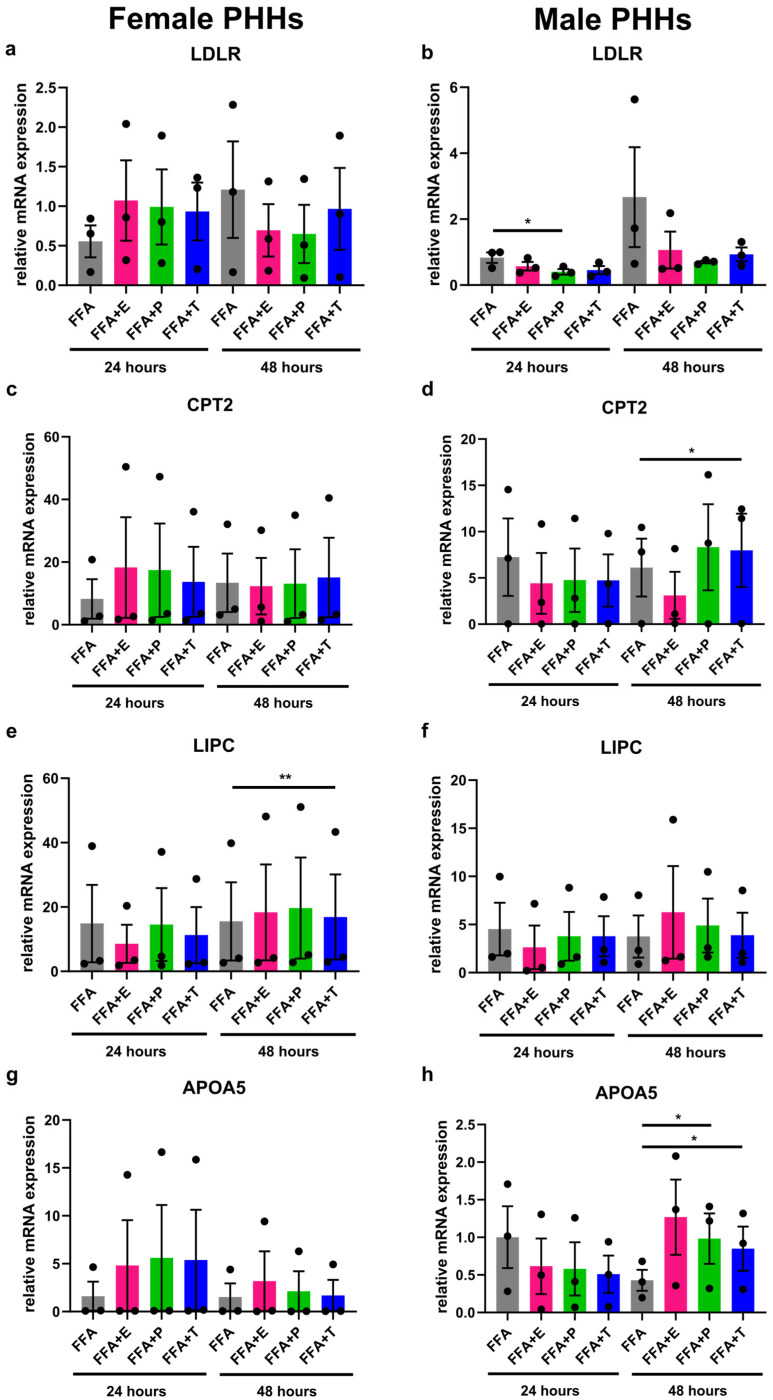
Influence of sex hormones on lipid metabolism gene expression in PHHs of different sex under steatotic conditions. Primary human hepatocytes (PHHs) from female and male donors were cultured with 0.6 mM free fatty acids (FFA) with or without addition of 10 nM 17b-estradiol (E), 70 nM progesterone (P), or 40 nM testosterone (T). mRNA expression levels of low-density lipoprotein receptor (*LDLR*), carnitine palmitoyltransferase 2 (*CPT2*), hepatic lipase (*LIPC*), and apolipoprotein A-V (*APOA5*) in female (**a**,**c**,**e**,**g**) and male (**b**,**d**,**f**,**h**) PHHs were analyzed by RT-qPCR. Individual relative gene expression values per donor are displayed as dots; bar graphs represent arithmetic means ± SEM. Statistical analyses were performed on log_2_-transformed expression values, *n* = 3 per sex, * *p* < 0.05; ** *p* < 0.01.

Under steatotic conditions, additional treatment with sex hormones led to changes in the expression of lipid metabolism genes that were different in male and female PHHs (Figure 4). Effects of testosterone and progesterone could be observed in particular. In male PHHs, apolipoprotein A-V (*APOA5*) and *CPT2* expressions increased significantly after 48 h with added testosterone in comparison to FFA treatment only. Likewise, progesterone increased the expression of *APOA5* after 48 h and further decreased the expression of *LDLR* after 24 h (and by trend after 48 h) under steatotic conditions. In female PHHs, FFA + testosterone for 48 h significantly increased the expression of hepatic lipase (*LIPC*). Further influences of sex hormone treatment on the expression of the analyzed genes in female PHHs could not be observed. Estrogen treatment only increased peroxisome proliferator-activated receptor alpha (*PPARA*) expression in male PHHs after 48 h (Figure S2 in Supplementary Materials). No effects of the combined FFA and sex hormone treatment could be observed for the gene expression of ATP-binding cassette, sub-family A, member 1 (*ABCA1*), apolipoprotein L2 (*APOL2*), and *PLA1A* (Figure S2 in Supplementary Materials).

### 3.5. Lipid Droplet Formation Is Sex-Specifically Influenced by Sex Hormones

**Figure 5:**
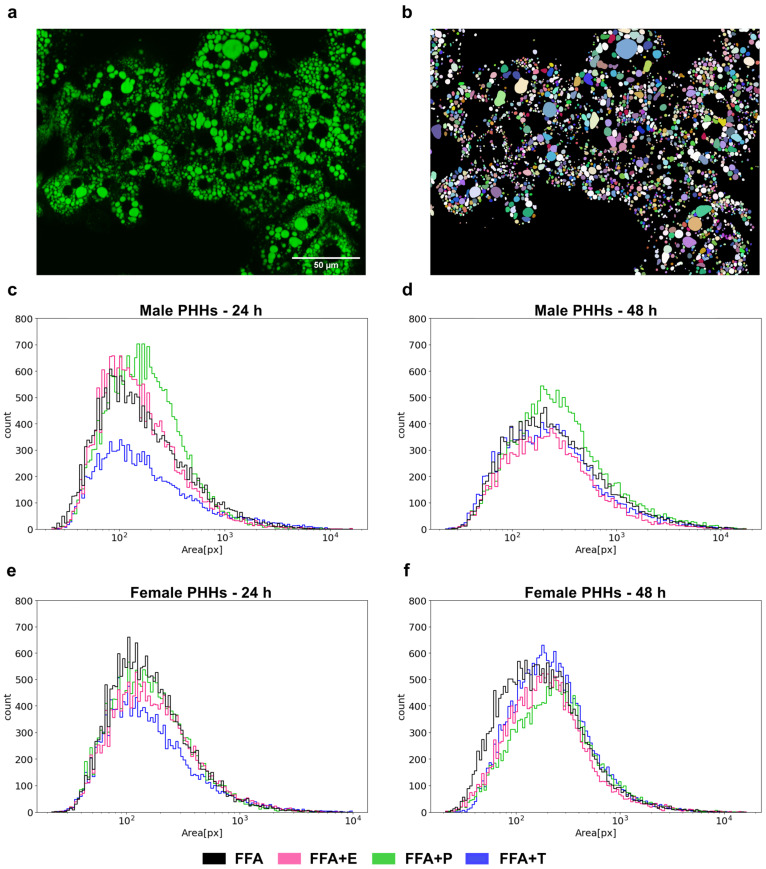
Sex- and sex hormone-dependent lipid droplet formation. Primary human hepatocytes (PHHs) of male and female origin were cultured with free fatty acids (FFA) with or without one of the three sex hormones 17b-estradiol (E), progesterone (P) and testosterone (T) for 24 h or 48 h. Lipid droplets were stained with BODIPY, and images were recorded with a Keyence microscope (**a**). LD areas were determined by imaging analysis (**b**) and are plotted as particle size distribution plots with the *x*-axis depicting the LD area in pixels and the *y*-axis depicting the LD count (**c–f**).

The formation of lipid droplets (LDs) was measured by fluorescence imaging in male and female PHHs after steatosis induction and additional sex hormone treatment (Figure 5). After 24 h of FFA treatment, female PHHs showed a more distinct left-shifted LD distribution with a larger accumulation of small-sized LDs, while the right-hand tail of the male LD distribution curve was more pronounced, displaying a relatively greater proportion of large LDs. After 48 h, the LDs enlarge in PHHs of both sexes, but the distribution pattern remains similar. In male PHHs, testosterone led to a marked decrease in LD size and count after 24 h, but the effect was diminished after 48 h. Under progesterone treatment, a marked right-shift in the LD distribution of male PHHs could be observed, indicating the formation of larger LDs. The hormone influence on the LD distribution in female PHHs was less distinct. But after 48 h, testosterone led to the formation of larger and estrogen of smaller LDs than FFA treatment alone. Also, progesterone induced a right-shifted distribution after 48 h in female PHHs, which was similar, but not equally pronounced as in the male hepatocytes.

## 4. Discussion

Our previous study on sex differences in PHHs revealed the importance of the consideration of cellular sex for investigations on the pathophysiological mechanisms of MASLD [14]. In the current study, we confirmed the necessity of integrating the variables of sex and sex hormones in in vitro studies modeling hepatic steatosis. It is, however, important to note that given the limited number of donors and inherent variability of primary human hepatocytes, the present findings should be regarded as exploratory. Nonetheless, the sex-specific patterns we observed are consistent with those of our previous work and clinical observations [9,10,11,14], thereby strengthening their biological plausibility and highlighting the need for larger confirmatory studies.

Steatosis induction in our model was achieved by application of oleate and palmitate in a 2:1 ratio based on the in vitro model of benign steatosis by Gómez-Lechón [17]. FFA overproduction in adipose tissue is the primary source of intrahepatic TAG in obese MASLD patients [24]. The model thus aims to reproduce the in vivo situation of FFA oversupply that causes MASLD. Our group has applied this approach several times before, thereby confirming a significant intracellular lipid accumulation without impairing hepatocyte viability [25,26,27]. Here, we expanded the model by introducing the variables sex and sex hormones. Regarding the source of primary cell isolation, it is important to state that the donors were carefully selected with respect to their comorbidities and medication. None of the donors had diabetes and none of them had lipid lowering medication.

The influx of FFA over the hepatocellular membrane occurs via a combination of passive diffusion (so called “flip-flop” mechanism) and facilitated transport by fatty acid transport proteins (FATPs) or fatty acid translocase (CD36) [28]. In rat hepatocytes, indications for a greater oleate transport rate over the plasma membrane of female cells was described, although the surface expression of fatty acid binding protein was identical between sexes [29]. Another report described higher *CD36* mRNA and protein expression levels in female rat livers and also slightly higher *CD36* mRNA levels in human livers of female origin [30]. Hepatic lipid contents in that study, however, did not differ between male and female rats, which is in line with our results showing similar intracellular TAG contents between female and male PHHs. Kinetic data on FFA influx in human hepatocytes is scarce and reports on sex-differences are lacking. We have recently reported long-term data of FFA uptake by PHHs that followed the sum of a saturable and non-saturable function as described by Bradbury and which was independent from baseline lipid content or donor sex [27,28]. Our current results confirm that in our model baseline FFA uptake and intracellular TAG accumulation do not differ between sexes. However, the number of excreted VLDL particles was twice as high in female than in male PHHs under steatotic treatment. In HepG2 cells, it was shown that oleate stimulates the secretion of apoB-100-containing lipoproteins by inhibiting the intracellular degradation of apoB [31]. Human in vivo studies demonstrated greater hepatic VLDL-TAG secretion rates and greater clearance of VLDL-TAG from plasma in lean women than in lean men [32]. Estrogen receptor α (ERα) was assigned an important role in modulating VLDL-TAG assembly and secretion and contributing to the observed sex differences in murine models. Hepatic ERα knock-out promoted hepatic steatosis and induced endoplasmic reticulum stress [33]. In our experiments, addition of estrogen did not lead to a further increase in apoB-100 secretion. But persistent sex-specific programming of the isolated PHHs could explain why female PHHs were more responsive to the FFA treatment and showed higher lipid excretion capacities than male ones. It could be assumed that increasing VLDL efflux might induce higher VLDL and LDL plasma levels and thus lead to a greater risk of atherosclerosis. This is, however, not the case in women, which is probably a consequence of higher VLDL plasma clearance rates of women in comparison to men [32]. One might therefore hypothesize that females show a greater flexibility to adapt their lipid metabolism to avoid hepatic steatosis without increasing the risk of atherosclerosis.

Addition of sex hormones in physiological concentrations unraveled further sex-dependent differences regarding lipid handling of PHHs. Regarding intracellular TAG accumulation, female hepatocytes benefitted from estrogen and male ones from testosterone application during short-term treatment of 24 h. The effects were diminished after 48 h, yet at that time point, testosterone treatment exacerbated lipid accumulation in female PHHs. In males, estrogen and progesterone also showed trends towards reduced TAG accumulation. However, these effects did not reach statistical significance. Our results underline clinical observations that point to a protective role for estrogen and opposite effects of testosterone in females concerning the development of hepatic steatosis, while males, again, seem to benefit from androgens [8,9,10,11]. Our observations were, for the most part, confirmed by our determinations of LD size distribution. As was recently reported in a diet-induced MASLD murine model, steatotic male PHHs contained greater amounts of large LDs than female ones [34]. This was also the case in our observations, especially after 48 h of FFA treatment. Under testosterone influence, LD formation in male PHHs was reduced in the short term, as was also observed for intracellular TAG content. This is in line with the results of a study applying a similar in vitro steatosis model in HepG2 cells, a hepatoma cell line of male origin. However, in that report, LD accumulation was also reduced by estrogen. But the applied hormone concentrations were more than 4 orders of magnitude greater than the physiological concentrations of our model [35]. In female PHHs, long-term testosterone treatment induced a right-shift in their LD distribution pattern, indicating an increased lipid storage in larger LDs, in line with the increased amount of TAG under the same conditions. These observations match a reported testosterone-induced increase in de novo lipogenesis in PHHs of female but not male origin [36]. Under progesterone influence, we observed a slight increase in female and a very pronounced increase in male lipid storage. In mice, progesterone has been shown to increase hepatic steatosis by inducing de novo lipogenesis [37], and in a cross-sectional study on patients with type 2 diabetes, progesterone levels were associated with the risk of MASLD in men but not in women [38]. Interestingly, progesterone also increased apoB-100 excretion in steatotic male PHHs. On the gene expression level, progesterone increased the mRNA levels of *APOA5* and decreased the expression of the LDL receptor gene (*LDLR*). APOA5, which is predominantly expressed by the liver, is a modulator of plasma triglyceride levels. APOA5 depletion has been suggested to promote hepatic steatosis in hamsters; however, reports on human APOA5 levels and the presence of MASLD render conflicting results [39]. Alongside progesterone, testosterone also increased *APOA5* in steatotic male PHHs, albeit to a lesser extent. Concerning LDLR, works on the primary hepatocytes of LDLR-disrupted male mice carved out a link between apoB-100 excretion and LDL receptor expression: the LDL receptor mediates both the degradation of apoB-100 before and after secretion and also its reuptake. In the absence of a functional LDL receptor, the rate of apoB-100 secretion increased [40]. Our results point in the same direction with a further influence of progesterone. The increased amount of apoB-100 in supernatants of progesterone-treated male PHHs may be a reflection of a decreased *LDLR* expression mediated by progesterone. These effects most likely underly a sexual dimorphism as they were not observed in female PHHs. Also, it remains unclear as to whether the link between *LDLR* expression and apoB-100 excretion underlies different regulatory mechanisms in females. In a former study, we analyzed the mRNA expression levels of eight lipid metabolism-associated genes that had been reported to show sex-biased expression according to transcriptomic studies [14]. A significantly higher *LDLR* expression in male than in female PHHs and a differential transcriptional reaction towards stimulation with sex hormones depending on hepatocellular sex was observed. In the current analysis, steatosis induction resulted in a significant upregulation of *LDLR* in male PHHs only. Furthermore, steatosis induction resulted in a significant upregulation of *CPT2* in male PHHs only. Apparent higher levels in females did not reach significance due to donor variability. Also, in male PHHs, *CPT2* expression increased even more upon FFA treatment paralleled by testosterone. A similar trend was observed under progesterone treatment, but this did not reach statistical significance owing to greater inter-donor variability. *CPT2* encodes for carnitine palmitoyltransferase 2, an enzyme located at the inner mitochondrial membrane, regulating fatty acid β-oxidation. CPT2 dysregulation may contribute to the development of MASLD and MASLD-associated hepatocellular carcinoma, which is why it has gained attention as a possible drug target [41].

Although female PHHs were influenced by sex hormones in their TAG accumulation and LD formation, little transcriptional changes were induced by either FFA treatment alone or in conjunction with sex hormones. The only significant response observed was a decrease in the mRNA expression level of hepatic lipase (*LIPC*) due to 48 h of FFA and testosterone administration. The activity of the lipolytic enzyme hepatic lipase is inversely correlated to high-density lipoprotein (HDL) levels and has long been known to be regulated in a sex-specific way [42], although the underlying mechanisms have still not been clarified. Sex hormones are probable contributing factors, but sex-specific differences in body fat distribution are also under discussion [43]. While most studies regarding hepatic lipase and sex differences focus on its protein activity, one study also reports sex hormone influence on its transcription: in HepG2 cells, estrogen treatment reduced *LIPC* mRNA expression [44]. In our current work, only minor estrogen-mediated effects on the expression of the analyzed genes could be observed.

This study has several limitations that need to be acknowledged. First, the selection of gene expression targets was guided by our previous work (Seidemann et al., 2024) rather than being systematically aligned with the functional readouts of hepatic lipid metabolism in the present study [14]. Second, we did not perform protein-level analyses, which would have been valuable to corroborate the observed transcriptomic changes. Third, because our gene expression analyses were not directly linked to functional alterations, mechanistic conclusions could not be drawn at this stage. In addition, the number of biological replicates was limited to three donors per sex. This constraint reflects the challenges inherent to studies with PHHs: each experimental setup requires large numbers of viable cells, and donor material is scarce and costly. We deliberately applied strict donor selection criteria (low BMI, absence of advanced steatosis, cardiometabolic health) to reduce confounding by comorbidities, but this further restricted availability. Importantly, all functional assays, imaging analyses, and gene expression measurements were performed with cells from the same donors, which allows for integrated interpretation of the data despite the small sample size. Nevertheless, the limited number of donors reduces the statistical power of this study and increases the influence of inter-donor variability. Our statistical analyses were therefore restricted to simple group comparisons, and the *p*-values are unadjusted. More advanced approaches such as mixed-effects modeling would require larger donor numbers and thus were not feasible in this study. We explicitly regard our findings as exploratory and hypothesis-generating, providing the rationale for larger confirmatory studies with expanded cohorts and more advanced statistical designs. Furthermore, our experiments were performed in the presence of 5% FBS, which may contain low levels of steroid hormones and binding proteins. While this could potentially affect free hormone availability, the use of FBS was necessary as a carrier for the FFA and to assure precise FFA concentrations to achieve reproducible steatosis induction in our model. Importantly, all experimental groups, including controls, were treated under identical conditions, and the hormone concentrations we applied were in the upper physiological range, far exceeding the trace hormone levels in FBS. Nevertheless, the use of FBS rather than charcoal-stripped serum is a limitation, and we will take this into account in the design of future studies. Despite these limitations, our study has important strengths, including its use of primary human hepatocytes, which are the gold standard for human liver metabolism studies; its systematic inclusion of both male and female donors; and its consistent application of experimental conditions across multiple assays. Building on this exploratory model, we plan to investigate lipid droplet remodeling in greater depth and to integrate transcript, protein, and functional analyses, thereby moving towards a more mechanistic understanding of sex-dependent lipid metabolism and its role in MASLD pathophysiology.

## 5. Conclusions

Our study demonstrates that sex and sex hormones significantly influence lipid handling in PHHs under steatotic conditions. Female PHHs displayed a greater capacity for VLDL particle secretion, while male PHHs showed stronger transcriptional responses. Sex hormones further modulated intracellular lipid accumulation and LD formation in a sex-dependent manner, with estrogen exerting rather protective effects in female hepatocytes and testosterone showing divergent effects depending on cellular sex. These findings support clinical observations of sex-specific differences in MASLD development and progression. While our data cannot provide mechanistic insights into the dysregulation of individual genes in MASLD, they emphasize the importance of considering sex-specific differences in hepatic lipid metabolism when studying disease pathophysiology and potential therapeutic strategies.

## Figures and Tables

**Table 1:** Donor data.

Donor	Sex	Age	BMI [kg/m^2^]	Steatosis ^1^	Diagnosis
FD1	Female	46	23	None	Sarcoma metastasis
FD2	Female	57	21	1%	iCCA
FD3	Female	65	22	None	Sarcoma metastasis
MD1	Male	46	22	None	Echinococcosis
MD2	Male	47	31	None	Chologenic abscess
MD3	Male	66	25	10%	CRLM

^1^ As reported in the postoperative pathohistological examination. Abbreviations: BMI, body mass index; iCCA, intrahepatic cholangiocellular carcinoma; CRLM, colorectal liver metastasis.

## Data Availability

The data presented in this study are available on request from the corresponding author.

## Supplementary Materials

The following supporting information can be downloaded at https://www.mdpi.com/article/10.3390/biomedicines13112770/s1: Figure S1: Cell activity of PHHs during cell culture under steatotic conditions and sex hormone treatment; Figure S2: Influence of sex hormones on lipid metabolism gene expression in PHH of different sex under steatotic conditions; Table S1: Primer specifications.

## Abbreviations

The following abbreviations are used in this manuscript:

ABCA1	ATP-binding cassette sub-family A member 1
APOA5	Apolipoprotein A-V
APOB-100	Apolipoprotein B-100
APOL2	Apolipoprotein L2
BCA	Bicinchoninic acid
BSA	Bovine serum albumin
CD36	Fatty acid translocase (Cluster of Differentiation 36)
CPT2	Carnitine palmitoyltransferase 2
DAPI	4′,6-diamidino-2-phenylindole
EEF2	Eukaryotic translation elongation factor 2
ERα	Estrogen receptor alpha
FFA	Free fatty acid
FBS	Fetal bovine serum
FATPs	Fatty acid transport proteins
HDL	High-density lipoprotein
HEPES	4-(2-hydroxyethyl)-1-piperazineethanesulfonic acid
LD	Lipid droplet
LDLR	Low-density lipoprotein receptor
LIPC	Hepatic lipase
MASLD	Metabolic dysfunction-associated steatotic liver disease
MEM NEAA	Minimum essential medium non-essential amino acids
NP40	Nonyl phenoxypolyethoxylethanol (Nonidet P-40)
PBS	Phosphate-buffered saline
PHHs	Primary human hepatocytes
PPARA	Peroxisome proliferator-activated receptor alpha
qPCR	Quantitative polymerase chain reaction
RPL13A	Ribosomal protein L13a
RPS18	Ribosomal protein S18
TAG	Triacylglycerol (triglyceride)
VLDL	Very-low-density lipoprotein
WME	William’s Medium E
