# Supplementary material for: Human Hepatocytes in Experimental Steatosis: Influence of Donor Sex and Sex Hormones
Source: Biomedicines. 2025 Nov 12;13(11):2770. doi: 10.3390/biomedicines13112770 (PMC12649905; doi:10.3390/biomedicines13112770)
Supplement: Supplementary file 1 [file biomedicines-13-02770-s001.zip › biomedicines-3895653-supplementary.pdf]

Supplementary Materials

Human hepatocytes in experimental steatosis: influence of donor sex and sex-hormones

Lena Seidemann [1,2], Carolin Marie Rohm [2], Anna Stilkerich [1,2], René Hänsel [3], Christina Götz [2], Daniel Seehofer [1,2] and Georg Damm [2,*]

Table S1. Primer specifications.

Gene	Assay ID[1] or primer sequence[2] (fwd / rev)
ABCA1	SBH0311739
CPT2	SBH0341291
LDLR	SBH0324029
PLA1A	SBH0212386
PPARA	SBH1220322
CYP3A5	SBH0140291
APOA5	tgggactacttcagccagac / cctcgctcccactcagag
APOL2	aggaccaagtgagcagagag / tgaccatgtgacttgcaagc
LIPC	gcaactctctcgaagccatg / tgctcccgggtaaaggtatg
UGT2B15	tcagtgtggacatcaggacc / tcgatccaggggcttcattg
SULT1A1	ctggagaagttcatggtcgg / gaggtagagaacagggtggg
RPL13A	cctggaggagaagaggaaagaga / ttgaggacctctgtgtatttgtcaa
EEF2	agaagctgtggggtgacag / gatcagctggcagaaggtg
RPS18	acatcgatgggcggcggaaa / ctcccgccctcttggtgaggt

[1]Primers purchased from Qiagen

[2]Primers purchased from Biomers

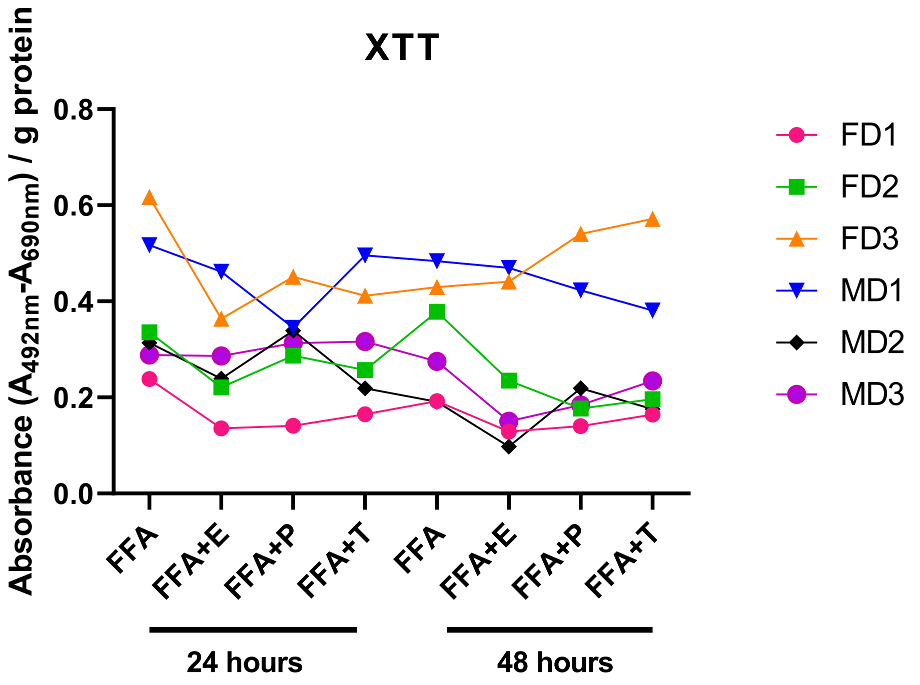

Figure S1. Cell activity of PHHs during cell culture under steatotic conditions and sex hormone treatment. Primary human hepatocytes (PHHs) from female and male donors were cultured with 0.6 mM free fatty acids (FFA) with or without addition of 10 nM 17b-estradiol (E), 70 nM progesterone (P), or 40 nM testosterone (T). Mitochondrial activity was measured by the conversion of XTT (2,3-bis-(2-methoxy-4-nitro-5-sulfophenyl)-2H-tetrazolium-5-carboxanilid) using Cell Proliferation Kit II by Sigma Aldrich (Merck KgaA, Darmstadt, Germany) according to the manufacturer's instructions. Absorbance was measured in two technical replicates with a microplate reader (Synergy H1, BioTek, Winooski, VT, USA) and normalized to total protein content determined by BCA assay.

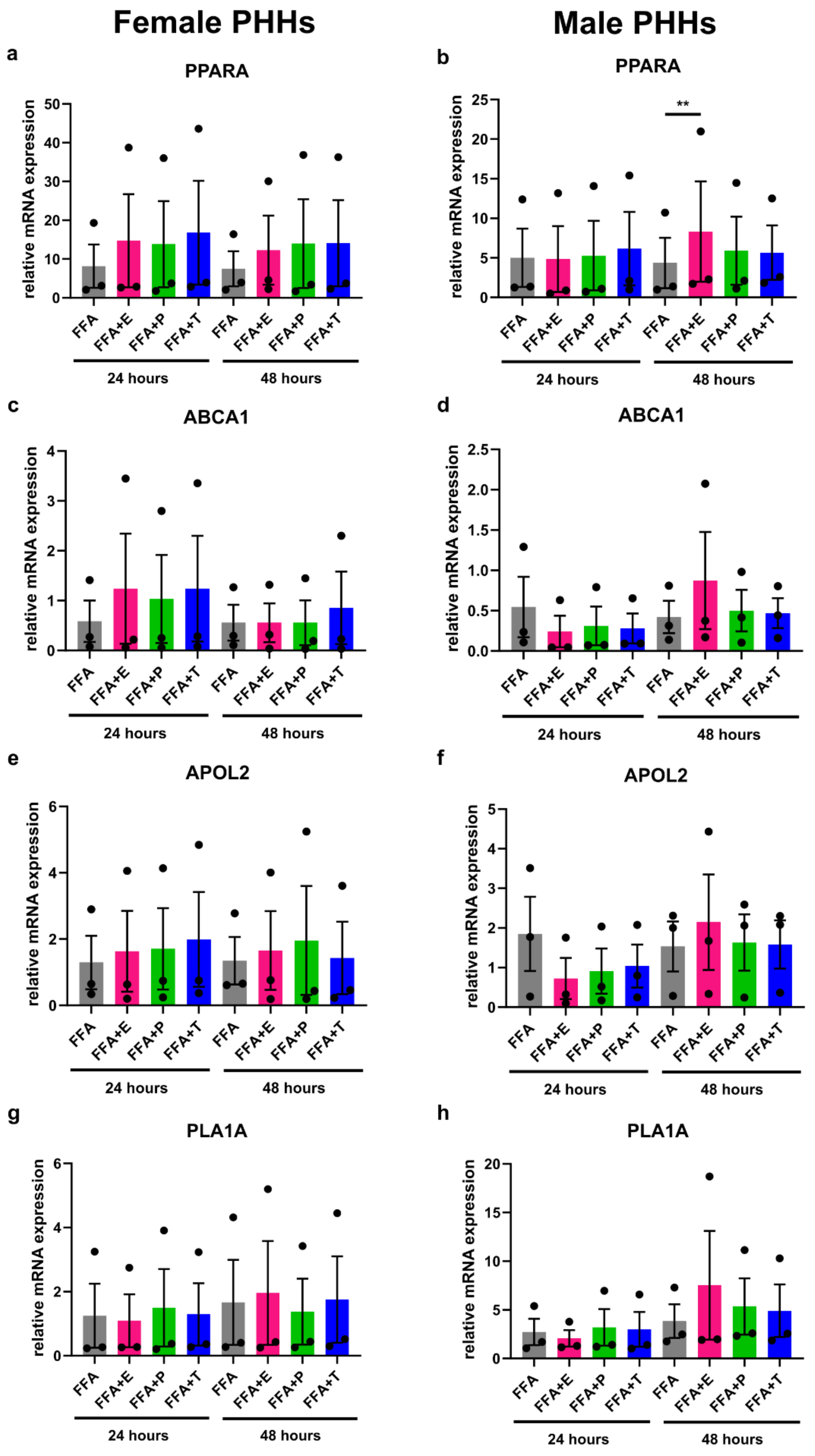

Figure S2. Influence of sex hormones on lipid metabolism gene expression in PHHs of different sex under steatotic conditions. Primary human hepatocytes (PHHs) from female and male donors were cultured with 0.6 mM free fatty acids (FFA) with or without addition of 10 nM 17b-estradiol (E), 70 nM progesterone (P), or 40 nM testosterone (T). mRNA expression levels of *PPARA* (peroxisome proliferator-activated receptor alpha), *ABCA1* (ATP-binding cassette, sub-family A, member 1), *APOL2* (apolipoprotein L2), and phospholipase A1 member A (*PLA1A*) in female (**a**, **c**, **e**, **g**) and male (**b**, **d**, **f**, **h**) PHHs were analyzed by RT-qPCR. Individual relative gene expression values per donor are displayed as dots, bar graphs represent arithmetic means ± SEM, statistical analyses were performed on log2-transformed expression values, $n = 3$ per sex, * $p < 0.05$; ** $p < 0.01$.
